# Epidemiology of Somatic Diseases and Risk Factors in the Population Living in the Zone of Influence of Uranium Mining Enterprises of Kazakhstan: A Pilot Study

**DOI:** 10.3390/healthcare11060804

**Published:** 2023-03-09

**Authors:** Elena Saifulina, Duisebai Janabayev, Yerlan Kashkinbayev, Aigerim Shokabaeva, Danara Ibrayeva, Moldir Aumalikova, Polat Kazymbet, Meirat Bakhtin

**Affiliations:** Institute of Radiobiology and Radiation Protection, Astana Medical University, Astana 010000, Kazakhstan

**Keywords:** uranium mining, epidemiological situation, risk factors, public health

## Abstract

The increase in uranium mining in Kazakhstan has brought with it a number of problems. Reducing the negative impact of radiation-toxic factors on the health of workers and the population in uranium mining regions is one of them. This article presents a pilot population health study in which we developed approaches to support residents living near an operating uranium mining enterprise. The purpose of the current study was to assess the impact of technogenic factors on the health of those living near the Syrdarya uranium ore province. Data collected from 5605 residents from the village of Bidaykol (the main group)—which is located 4 km from the uranium mining enterprise—and the village of Sunakata (the control group), which is located in the Kyzylorda region, were analyzed. A bidirectional cohort study was conducted. The prevalence of acute and chronic diseases among the residents of Bidaykol was 1.3 times higher than that in the control group. The structure of morbidity was dominated by pathologies of the genitourinary system (27%), the circulatory system (14.4%), and the respiratory system (11.9%). Relative risks (RR > 1) were identified for most classes of diseases, the highest being for diseases of the blood (RR = 2.6), skin (RR = 2.3), and genitourinary system (RR = 1.9). In the main group, renal pathologies were the most frequent class in the age group of 30–40 years, occurring mainly in women. In addition, they had a direct dependence on the duration of residence in the territory of the uranium ore province. Further studies into the interaction between the technogenic factors associated with uranium mining enterprises and the development of diseases of the urinary system are needed. This will make it possible to determine ways to prevent these pathologies in the population.

## 1. Introduction

Kazakhstan has rich natural resources such as minerals, metal ores, natural gas, and oil reserves. Uranium mining in the Republic of Kazakhstan started in 1940. The mining and milling of uranium ore in North and South Kazakhstan have caused environmental contamination through a number of activities, specifically, the open-pit mining process, transportation to and from milling sites, the milling and processing of ore, and the open-air storage of radioactive and nonradioactive mining wastes [1].

Ecologically determined pathologies are especially problematic in the uranium mining regions of Kazakhstan, which include the Syrdarya uranium ore province [2]. As in most uranium mining enterprises in the Republic, the method of in situ leaching is used. During the extraction and processing of uranium ores, a significant amount of chemically active substances is released into the environment [3]. During in situ leaching, pollution is mainly associated with the impact of technogenic radionuclides, which are harmful to human health and wildlife on Earth’s surface and in its aquifers [4]. The medical consequences of anthropogenic environmental pollution are a state policy priority; however, there are few studies devoted to risk assessments in populations living in areas close to uranium mining enterprises.

The growth in uranium production has a negative impact on the environment and the nearby population. A previous study of the radiation situation in the Syrdarya ore province assessed gamma radiation levels beyond the sanitary buffer zone of the Northern Karamuryn uranium deposit and the uranium concentration in water samples. The authors concluded that the environment in adjacent settlements was being contaminated [5].

Various studies investigated the effect of chronic exposure to low-level radiation on human health in settlements located near the Syrdarya uranium ore province. The results showed an increased prevalence of kidney pathologies among residents in the observed area [6]. 

This study is a pilot study that involves preliminary data on the prevalence of diseases in a population that has been living near an operating uranium mining enterprise for a long period. The results of this study can help determine whether there is a relationship between the radioecological situation in settlements and the health status of their inhabitants. In the future, this will help to identify measures that ensure the radiation safety of the population.

The study’s aim was to assess the impact of technogenic factors associated with uranium mining enterprises on the health of those living near the Syrdarya uranium ore province.

## 2. Materials and Methods

This study of the health status of the population living in the zone of influence of operating uranium mining enterprises was conducted within the framework of the project “Development of methods for leveling negative technogenic risk factors for the environment and health of the population of the Syrdarya uranium ore province” (No. 158/36-21-23 from 27 April 2021, IRN AP09261243). 

This research was aimed at addressing a number of problems related to managing radioecological risks. It provides a framework for the development of a system of rehabilitation measures to mitigate the consequences of radioactive environmental contamination and reduce the radiation risks to the population as a result of the activities of uranium mining enterprises.

**Figure 1:**
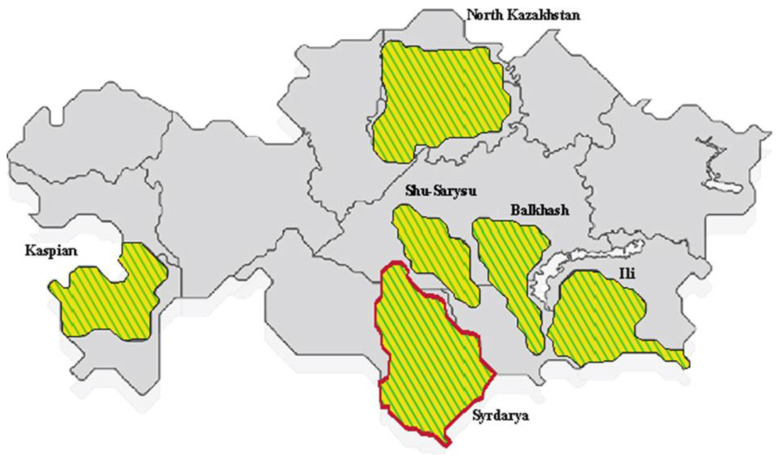
Uranium provinces of Kazakhstan, indicating the distribution of uranium reserves: Syrdarya (10.2%); North Kazakhstan (13.1%); Shu—Sarysu (66.8%); Ili (6.1%); Caspian (3.1%); Pribalkhashskaya (0.7%) [7].

The main uranium deposits located on the territory of the Republic of Kazakhstan are located in six uranium ore provinces (Figure 1).

One of the largest amongst these is the Syrdarya uranium ore province, located in South Kazakhstan, which was discovered in the 1970s and 1980s [8]. The Syrdarya uranium ore province includes 9 large uranium deposits and uranium mining enterprises are located on its territory near various settlements.

The object of our study was the population of the village of Bidaykol, which is located close to the uranium mining enterprise, i.e., 4 km away. Preliminary studies of the radiation situation in settlements located near active uranium deposits were conducted. In the soil samples from Bidaykol, increased specific radionuclide activity was recorded: Ra 226 and Th 232, which were as much as 5 times and 4 times the average values for the country, respectively. In water samples taken from wells in the Bidaykol settlement, the total alpha activity was as much as 3 times higher than the control values. In water samples from a 12-m-deep well in Bidaykol, the concentration of uranium was as much as 2 times that of the maximum permissible concentration [9]. In the territory of Sunakata, which was selected as a control, the radiation situation was stable, and the radiation background was within the background level. The specific radionuclide activity of the uranium and thorium series in water and soil samples corresponded to the average values for the country.

In order to study the possible impact of the radiation situation on the health of the population, a bidirectional cohort study was conducted, which included 5605 rural residents from the Kyzylorda region in the Republic of Kazakhstan. The cohort was established in 2021. Participants were tracked retrospectively, i.e., from 2010 to 2021, and prospectively, i.e., studies are due to continue until 2023, in the same way for both groups. The studied cohort included the population of Bidaykol; Shieli district, as the main group; and residents from Sunakata, Zhanakorgan district, the Kyzylorda region, located more than 15 km from the nearest uranium mining enterprise as a control group. 

In the first stage of the pilot epidemiological study, outpatient medical records were collected in polyclinics and inpatient medical records in district hospitals. Statistical records of people who left the hospital and data on the mortality of the population in the studied settlements were also obtained. According to the data from regional executive authorities (akimats), in 2021, 4287 people lived in Bidaykol, and 1956 people lived in Sunakat. Moreover, in our study, the coverage of residents in the Bidaykol and Sunakata settlements was almost complete: the main group comprised 3754 (87.6%) individuals and the control group comprised 1851 (94.6%). The entire population could not be included in the study due to certain individuals using other polyclinics, for example, students studying in cities, and residents who did not seek medical help and who had not undergone medical screenings. In addition, the main selection criterion for the study groups was a long period of residence in these territories, i.e., more than 15 years. According to outpatient cards, the date of registration in the territorial polyclinic and the person’s previous location were tracked. The exclusion criterion was professional contact with sources of ionizing radiation, e.g., working at a uranium mining enterprise. These residents were also not included in the study. The distribution of the population by age and sex is presented in Table 1.

The study on the prevalence of diseases was mainly conducted among the adult population. There was no statistical difference between the adult population of Bidaykol and Sunakata by age (*p* = 0.93) or gender (*p* = 0.075). The studied territories are classified as rural areas in terms of their social and industrial infrastructure, i.e., the population leads a traditional way of life. The control group was selected due to the similarity in natural, climatic, and social conditions as the exhibited group, but the different environmental radiation situation. 

In the second stage of the study, the medical data of the population from the main and control groups were entered into an electronic database, i.e., the sectoral radiation and epidemiological register of the Institute of Radiobiology and Radiation Protection of NAO “Astana Medical University”. This register allows for the dynamic monitoring of the population living in ecologically unfavorable areas. The data entered into the electronic database are presented in Table 2.

A comprehensive study of the influence of environmental factors on the health of the population must necessarily take into account the principle of “feedback”.

In this regard, a questionnaire was developed that contains 10 questions to determine the attitude of the respondent to the environmental situation in the region with a self-assessment of their own health. The population survey was simultaneously conducted using a cross-sectional research method in all groups in the summer of 2022. Respondents filled out the questionnaire on their own in the presence of the person responsible for conducting the survey. To do this, a sample was formed from the adult population from the studied settlements. The main group of respondents comprised 330 people from Bidaykol (a sample from the adult population of the main group with a confidence level of 95%). At the time of study, all of them had lived for a long period in the territory of the village, i.e., in the zone of influence of the uranium mining enterprise. At the same time, the survey was conducted in the control group from Sunakata. This group comprised 292 people.

When analyzing the results of the study, relative risks (RR) were calculated for all classes of diseases. To compare the two samples, nonparametric tests were used: the Mann–Whitney U test and the binomial test. Categorical variables were compared using a nonparametric χ^2^ test. A direct standardization method was used to compare disease prevalence by age and gender. The analysis was performed using the IBM SPSS Statistics 20 software and Microsoft Excel.

## 3. Results

According to the results of a survey of the adult population of Bidaykol, the vast majority of respondents regard the environmental situation in the village as unfavorable. Similarly, in the control group, most of respondents answered in the same way. The Bidaykol group highlighted the following environmental features as characteristic of their region: air pollution with dust, the presence of hazardous industries in the region, poor quality drinking water, etc. The control group highlighted air pollution with dust and the presence of hazardous industries in the region as problematic. Respondents from both groups were concerned about living in a territory with harmful industrial enterprises. In the village of Bidaykol, residents highlighted air pollution. Those from Sunakata were concerned about the poor quality of drinking water. The results of the survey concerning the ecological situations in the two regions are presented in Table 3.

In total, 30% of respondents had land within the boundaries of the settlement on which they grew vegetables and fruits. In addition, the majority of respondents from Bidaykol reported eating food grown in the area (55%). That is to say that the majority of respondents consumed agricultural products grown in contaminated areas. Similar results were found for products of animal origin (meat, milk, eggs, fish). Most respondents (60%) kept livestock, which also grazed in areas close to the uranium mining enterprise. Moreover, 38% of residents who did not own a household chose only local products. Only 2% of respondents reported eating imported products of animal origin.

The health status self-assessment of residents from Bidaykol shows that the majority of respondents (47.5%) regarded their health as “good”, and 37.5% as “satisfactory” or “bad”. The rest found it difficult to answer. In the settlement of Sunakata, 51.3% of respondents considered their health to be “good” and 41% as “satisfactory”. Nevertheless, the vast majority of respondents from Bidaykol (52.5%) directly associated health problems with environmental pollution. This was 48.7% in the control group. The health self-assessment scores among residents from the main and control groups were statistically insignificant. Therefore, it can be assumed that, in the case of illness, accessibility to medical care in both populations was approximately equal. 

The most informative and reliable criteria of public health adopted by WHO are medical and demographic indicators. According to their size and dynamics, conclusions are usually drawn about the epidemiological well-being of the population and the potential for its further development.

A data analysis of the entire population, including adults and children, showed that the prevalence of acute and chronic diseases was higher among residents of Bidaykol, which amounted to 1745.1 per 1000 people, whereas, in the control group, the prevalence of diseases was lower, i.e., 1257.7 per 1000 people. Genitourinary system pathologies were the most common pathology in main group (21.4%), with a significant prevalence of tubulointerstitial kidney diseases. Respiratory diseases were the second most frequent (18.1%), and digestive diseases ranked third (12.0%). In the control group, respiratory system diseases were most common (22.8%), followed by genitourinary system diseases (17.5%), and circulatory system diseases (13.6%).

**Figure 2:**
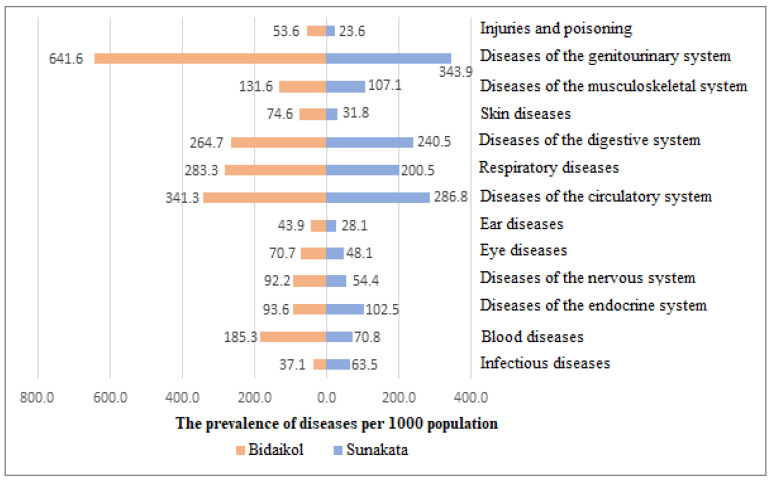
Comparative characteristics of the prevalence of diseases in the studied groups (per 1000 people).

When analyzing disease prevalence in the adult population (over 18 years of age) in the main group, i.e., those living near uranium enterprises, it was found that in most classes of diseases according to ICD-10, prevalence exceeded that in the control group, i.e., those living further from the uranium mining industry (Figure 2). In addition, diseases of the genitourinary system were the most common (27%), i.e., 641.6 per 1000 people in Bidaykol, almost double the value observed in the control group (343.9 per 1000 people, χ^2^ = 255.6, *p* = 0.00). These were followed by diseases of the circulatory system (14.4%), and then by diseases of the respiratory system (11.9%). In the control group, genitourinary system diseases were also most common, followed by diseases of the circulatory system and digestion. The exceptions were infectious diseases and diseases of the endocrine system, which were more common in the control group; however, the differences between the groups were statistically insignificant.

**Figure 3:**
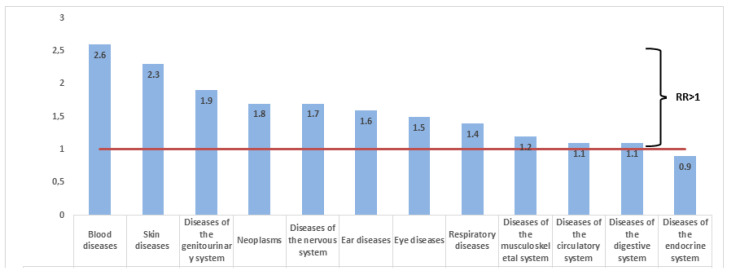
Relative risks of somatic diseases in the population from Bidaykol according to the ICD-10 classes.

To determine the risk of diseases in the main group in relation to the control group, a calculation of relative risks (RR) according to ICD-10 classes was conducted. The highest RR was found for blood diseases, i.e., 2.6 with a CI of 2.0–3.3. Among the diseases of the blood system, anemia was most common at 17.4 per 100 people. For skin diseases, RR = 2.3, CI 1.6–3.5, and for diseases of the genitourinary system, RR = 1.9, CI 1.7–2.1 (Figure 3).

A comparative assessment of the prevalence of diseases in the studied groups by age was conducted in order to identify “at-risk” age groups (Table 4). No pronounced patterns were found when comparing the frequency of disease occurrence in the main and control groups for the prevailing classes of diseases; however, in general, statistically significant differences were found in older age groups for all diseases included in the ICD-10. In the context of certain classes of diseases, this trend was particularly clear for diseases of the circulatory system in the Bidaykol group, where the prevalence of diseases per 1000 people increased from 87.4 at the age of 20–30 years to 1130.4 in people over 70 years (χ^2^ = 316.3, *p* = 0.00).

Diseases of the genitourinary system, on the contrary, were common in all age groups, with a peak at 30–40 years, i.e., 757.3 per 1000 people. 

After direct standardization by age, we obtained the following indicators of disease prevalence: in the main group, the highest was in the age group of 30–40 years (the expected result was 543 per 1000 people); in the control group, the highest was in the age group of 40–50 years (the expected result was 315.6 per 1000 people). Thereafter, the standardized incidence ratio (SIR) for diseases of the genitourinary system was calculated, producing the following results: 1.9, CI 1.7–1.9.

Statistically significant differences were found for diseases of the genitourinary system according to gender: among women, the prevalence was two times higher and amounted to 892.0 versus 427.8 per 1000 people. Diseases belonging to the category of “chronic tubulointerstitial nephritis” prevailed in both sexes. Direct standardization of prevalence rates of tubointerstitial kidney disease by sex showed that high values of this indicator (two times higher) were also recorded for females, both in the main and in the control group.

In general, the frequency of disease occurrence of the prevailing classes (genitourinary, cardiovascular, and respiratory systems) by gender demonstrated a preponderance for the female sex, both in the main and in the control group. In addition to age and gender, an analysis of the prevalence of kidney diseases was conducted depending on the duration of residence in the zone of influence of uranium mining enterprises: no kidney diseases were registered at all in the adult population living in Bidaykol for up to 5 years and from 5 to 10 years. However, the prevalence increased with an increase in the period of residence in the territory as follows: 10–20 years—76.9; 20–30 years—406.9; and more than 30 years—366.7 per 1000 population.

Thus, the results of the conducted studies showed a high prevalence of diseases of the urinary system among residents of Bidaykol as compared with the control group. They also demonstrated a dependence of the frequency of kidney disease occurrence on age, gender, and duration of residence in the study area.

## 4. Discussion

Kazakhstan is the largest and richest country in Central Asia in terms of resource potential. The Republic has significant oil, gas, coal, and uranium reserves—the extraction and export of which have ensured and, in part, still ensure the rapid growth of the economic well-being of the country and its citizens. The total explored uranium reserves in the country are estimated to be more than 9,068,700 tons. Since 2009, the Republic has ranked first in uranium mining in the world, producing about 41% of the world’s uranium [10,11]. Thus, according to Kazatomprom, the proceeds from the sale of uranium products in Kazakhstan in 2021 amounted to KZT 625,048 million for the 21,819 tons produced, and this figure is growing from year to year [12].

The underground borehole leaching method is a modern and cost-effective method of uranium mining. Industrial uranium production in Kazakhstan has been entirely conducted using the ISL method since 1998 [7]. This method involves developing the ore deposits without raising them to the surface by selectively transferring natural uranium ions into a productive solution directly in the earth. The opening and extraction of uranium is conducted by leaching through wells drilled from the surface [13].

Uranium mining via underground leaching plays an important role in global uranium production. However, this technology can be ecotoxic and have a serious impact on both the environment and human health [14,15]. When uranium is mined using this method, groundwater and soil can be contaminated with natural and man-made radionuclides. In addition, it is known that groundwater contamination can persist for long periods even after the extraction of uranium from the production zone [16]. Moreover, a number of studies have shown that the restoration of groundwater to the concentrations before mining with the use of ISL is a difficult task [17,18].

In addition to groundwater, soil pollution can also occur during ISL. This can result from the leakage of solutions from damaged pipes or the pumping undertaken for cleaning or sampling. Sulfuric acid and its salts, nitrates, and radionuclides (uranium, thorium, radium, polonium, etc.) can penetrate into the earth’s surface with solutions. As a result, the soil may temporarily become unsuitable for plant life, or these plants can acquire properties that are dangerous for animals and humans [19].

The problem of environmental pollution with natural and man-made radionuclides is relevant for many countries; however, it is especially acute in countries in the former Soviet Union, including Kazakhstan [20]. In many post-Soviet countries, uranium industry enterprises and radioactive waste storage facilities were located near settlements and along the paths of large rivers flowing into the densely populated valleys [21]. The Syrdarya River is the main artery for large agricultural and industrial areas in the Turkestan and Kyzylorda regions of Kazakhstan. Approximately 200 settlements and more than 90 industrial enterprises are located along the banks of the Syrdarya River. The uranium mining enterprise of NAC Kazatomprom JSC is located in the Shieli district, the Kyzylorda region in mining department No. 6, which was established in 1983. The settlements of Bidaykol, Akmaya, and Shieli are nearby, which are named after Zhakaev and others [22].

In order to identify the negative impact of unfavorable technogenic factors on medical indicators of human health, it is necessary to have reliable information about anthropogenic environmental pollution and the ecological state in the territories in uranium regions. It is known that radionuclides can enter the human body by inhalation, orally, and through the skin. However, for people living in areas contaminated with radionuclides, the enteral route is of greater importance. The population may be chronically exposed to radionuclides through drinking water or food [23,24,25].

The supply of uranium to humans in food is also important. The main food chains are as follows: plants→human; plants→animal→milk→human; plants→animal→meat→human; plants→bird→egg→human; water→hydrobionts→human [26].

As the survey showed, the environmental situation in the settlement located near the uranium mining enterprise is of concern to local residents. The majority assessed the ecological situation as unfavorable, naming the proximity of industrial enterprises among the main negative factors in the area. The results of the study show that there is a high risk of eating agricultural products grown in contaminated areas and local animal products.

Therefore, all the described scenarios of enteral radionuclide intake can occur in the region under study. Once inside the human or animal body, transuranium radionuclides can be absorbed into the blood and enter the parenchymal organs. The deposition of uranium and its daughter radionuclides in organs and tissues can last from several days to several years, which may cause unpredictable negative health effects [27,28]. The accumulation of uranium mainly occurs in the bones, kidneys, and liver [29]. According to recent studies, uranium primarily causes pathological changes in the body through toxic effects in the kidneys, bone tissue, liver, reproductive system, lungs, and nervous system [30].

Assessing the health status of residents living near active uranium deposits, one can see that the prevalence of somatic diseases and the structure of morbidity differ from residents living in radionuclide-free regions. Thus, the prevalence of diseases of all classes in the population from Bidaykol was 1.3 times higher than those from the control settlement (the village of Sunakata). This pattern was observed both among the entire population and among the population over 18 years of age.

Since the main selection criterion for the study groups was having resided in the studied settlements for more than 15 years, a pilot epidemiological study was conducted in the adult population.

In regard to the structure of morbidity, the most common groups were diseases of the genitourinary system and cardiovascular and respiratory diseases. The sum of the proportion of these three classes accounted for more than half (53.3%) of all disease cases. A slightly different picture was observed in the control group: there was a high proportion of diseases of the digestive system. Thereafter, intensive indicators (frequencies) of morbidity among workers in the main and control groups were calculated. This made it possible to determine the statistical significance of differences among those living in the uranium mining province for most classes of diseases (Figure 1). Relative risks (RR > 1), which can indicate that living conditions near the uranium industry are a risk factor for human health, were also identified for almost all classes of diseases (Figure 2). The highest RRs were characteristic of diseases of the blood, skin, and genitourinary system. The study of the long-term consequences of radionuclides in the blood system remains an important topic in biology and medicine. It is known that radionuclides significantly affect the hematopoietic system by reducing leukocytopoiesis [31]. In our study, the largest proportion assigned to the structure of morbidity was that of anemia (iron deficiency or unspecified). This, once more, calls for a more in-depth analysis and laboratory studies focused on the inhabitants of Bidaykol and the causes of disease in the area. Dermatitis and eczema were the most common skin diseases in the main group, which could also hypothetically be the result of water and soil pollution by heavy metals from uranium mining deposits.

On the basis of the results, a comparative analysis of the radiation situation and the state of health of the population living near the uranium deposits in the Syrdarya uranium ore province (the southern region of the country) and the Stepnogorsk region in the North Kazakhstan uranium province was conducted. In the Stepnogorsk region of Northern Kazakhstan, a uranium processing industry enterprise is currently operating, and there are depleted uranium mines and a radioactive waste storage facility. Studies of the radiation situation in the territories of the Zavodskoy, Aksu, and Kvartsytka settlements made it possible to establish contamination levels with technogenic radionuclides of the uranium and thorium series in these areas and problems related to radon safety. The concentration of radon exceeded the maximum permissible level in residential areas of settlements by as much as two times, with it exceeding the maximum permissible level by 10 times in a particular secondary school. For drinking, the population used imported water that meets sanitary and hygienic requirements [32]. The results of pilot epidemiological studies in the uranium regions of Southern and Northern Kazakhstan exhibited significant differences. Among the population of the Stepnogorsk region, diseases of the circulatory system, the musculoskeletal system, and respiratory diseases were common [28,33]. Long-term low-level exposure may have caused the high prevalence of diseases of the cardiovascular system, which exists alongside the traditional risk factors [34]. The accumulation of gaseous radon in residential premises is a cause of respiratory pathology in the population [35].

We believe that the high prevalence of diseases of the genitourinary system in Bidaykol may be associated with contamination with radionuclides and heavy metals in water sources, which are used by residents both as drinking water and for irrigating cultivated plants and watering livestock. Laboratory analyses of drinking water samples taken from wells in Bidaykol showed that the total alpha activity was as much as four times higher than the permissible level. Elevated concentrations of chlorides and cadmium in drinking water and elevated concentrations of chlorides, sulfates, cadmium, nickel, and chromium in the Syrdarya River in South Kazakhstan suggest water pollution due to the cultivation, processing, and storage of rice. Collector-drainage waters from rice fields were the main sources of pollution in this region [36].

An analysis of the data showed that the main diseases in the region under study were diseases of the genitourinary system. Moreover, the subclass “tubulointerstitial kidney disease” accounted for 80% of all MPS diseases. In the medical records of outpatients with kidney disease, the final diagnoses were either chronic pyelonephritis or the subclass name was duplicated, i.e., tubulointerstitial kidney disease.

However, the relationship between possible exposure to uranium from the Bidaykol environment and impaired renal function remains unclear. A high prevalence of kidney disease was found in all age groups, with a peak at the age of 30–40 years. Diseases of the urinary system among women were twice as common. It is known that pyelonephritis exceeds all renal diseases combined in terms of frequency and is among the most common diseases of the urinary system associated with infection. However, according to statistics, in young women, pyelonephritis occurs 5–7 times more often than in men.

Nearly one in three women will have had at least one episode of UTI requiring antimicrobial therapy by the age of 24 years. A progressive increase in the prevalence of pyelonephritis among men only occurs in the elderly and those who are senile. By this period, the functional activity of the prostate gland decreases, the protection of the urinary tract decreases, and the frequency of hypertrophic and tumor processes in the prostate increases. This leads to impaired urodynamics, which, together with microbial invasion, leads to pyelonephritis [37].

However, in our study, the difference in the prevalence of tubulointerstitial diseases among young men and women was not as pronounced. An in-depth analysis of this category of kidney diseases and an investigation into the reasons for the relatively high prevalence in men are required. In addition, it is necessary to establish whether the majority of kidney diseases in the population from Bidaykol are of an infectious nature or are tubulointerstitial nephritis. Tubulointerstitial nephritis is characterized by a heterogeneous group of acute or chronic abacterial, nondestructive lesions of the renal tubules and interstitium, with the spread of the inflammatory process to all structures of the renal tissue [38]. One of the nosological forms of tubulointerstitial kidney damage is tubulointerstitial and tubular lesions caused by drugs and heavy metals [39]. These forms of renal pathologies may result from living in the zone of influence of uranium mining deposits, as it is known that uranium has a toxic effect on the kidneys both as a heavy metal and as an alpha-emitting radionuclide. The mechanism of action of uranium and transuranium radionuclides on the renal tissue is the deposition of uranyl ions in the epithelium of the tubules, which results in damage [40,41].

Given the nephrotoxicity of uranium, synergistic effects causing renal pathology development among populations living near uranium enterprises cannot be ruled out. This effect may also be exacerbated by additional exposure to toxic substances. Thus, synergistic health effects need further study.

In addition, it was found that the prevalence of renal pathologies increased with the increase in the period of residence in the territories under study. Thus, this is an additional risk factor for the populations in the studied settlements. 

Our study had a number of limitations. Firstly, there is the lack of official statistics on the settlement scale. Such data are presented only in the regional context in the Republic. In addition, for automation in medical organizations in Kazakhstan, the Integrated Medical Information System (IMIS) software was introduced. However, in small settlements, it has only been operating since 2020. In this regard, a great deal of the retrospective medical data were not correct, especially those regarding the date and staging of newly diagnosed diseases. Accordingly, it was not possible to conduct an analysis according to the level of morbidity. Thus, our study refers to the prevalence of diseases.

Secondly, the disease prevalence was higher among the residents of Bidaikol in almost all classes of diseases, with the exception of infectious and endocrine pathologies. There is a possible reporting bias since almost every disease category in the exposed group was increased. Chronic radiation-chemical exposure to ionizing radiation does not have such an extensive effect. Therefore, there may be differences in the quality of medical care or medical records in the villages of Bidaykol and Sunakata, or there are additional risk factors among the residents of the village in the main group.

Despite the limitations of our pilot study, we were able to identify a pathology that is typical for residents of Bidaykol. It is probable that the radioecological situation in the region played a significant role in the high disease prevalence in various organs and systems, especially genitourinary diseases.

Research in this direction will continue. Further studies of the relationship between environmental distress and the development of diseases of the urinary system will make it possible to determine ways to prevent this pathology among populations living in areas near to uranium mining enterprises.

Ultimately, it is necessary to develop a set of measures that mitigate the radiation risk for the population living near the uranium deposits in South Kazakhstan. In order to strengthen measures that maintain the concentration of radionuclides within acceptable limits, purposeful communication between interested state bodies, scientific and medical organizations, heads of uranium mining complexes, and local authorities is of great importance.

## 5. Conclusions

Thus, the results of this pilot epidemiological study demonstrate a higher prevalence of diseases among residents in settlements located near the active uranium deposits in the Syrdarya uranium ore province. The residents of Bidaykol were found to have a high prevalence of diseases of the genitourinary system, and circulatory and respiratory systems. Tubulointerstitial kidney diseases were most common among genitourinary system pathologies. In this work, it was shown that the influence of radiation and chemical factors on the occurrence of renal pathologies in people living for long periods near a uranium mining enterprise cannot be excluded. An in-depth study of factors that affect the high prevalence of kidney disease is needed. The concentration of heavy metals in drinking water and uranium in the urine of residents from Bidaykol and Sunakata will be obtained in future studies. In the next stage, we plan to study medical data from the population with genitourinary system pathologies, combining urine test results and kidney ultrasound data.

## Figures and Tables

**Table 1:** Characteristics of the population of the main and control groups.

Characteristics/Settlement	Bidaykol Village	Sunakata Village
Total population (2021)	4287	1956
Number of people in study groups	3754	1851
Under 18 years old	1703 (45%)	749 (40%)
Older than 18	2051 (55%)	1102 (60%)
Adult population:		
Average age	43 ± 16	44 ± 16
Female	1102 (54%)	524 (48%)
Male	949 (46%)	578 (52%)

**Table 2:** Data of the population living in the Syrdarya uranium ore province.

Data Groups	Basic Information
Personal data	last name, first name, patronymic, date, and place of birth
Data on the place of residence	length of residence in the given locality, exact address
Professional activity	place of work, profession, and length of service
Social factors	smoking, alcohol consumption
Information about morbidity	date of detection of the disease, disease code according to ICD-10
Medical data of screening medical examinations	height, weight, blood pressure, pulse, date of examination, and results of medical examination
Clinical examination	information regarding hospital registration

**Table 3:** The results of the survey on the ecological situation in the region.

Study Question	Bidaykol	Sunakata
How do you assess the ecological situation in the area where you live?		
favorable	30%	28.2%
unfavorable	67.5%	69.2%
find it difficult to answer	2.5%	2.7%
List the features of the environment characteristic of the region where you live:		
adverse climatic conditions	9.6%	8.5%
poor drinking water quality	15.4%	31.9%
dust air pollution	32.7%	23.4%
pollution of water bodies and soil with waste	11.5%	4.3%
the presence of hazardous industries in the region	30.8%	31.9%
How would you rate the state of your health?		
excellent	5%	5.1%
good	42.5%	51.3%
satisfying	25%	41.0%
bad	12.5%	-
find it difficult to answer	15%	2.6%
Are there health problems associated with environmental pollution?		
yes	52.5%	48.7%
no	22.5%	20.5%
find it difficult to answer	25%	30.8%

**Table 4:** Prevalence of prevailing classes of diseases by age categories of the Bidaykol group.

Name of Disease Classes According to ICD-10	20–30	30–40	40–50	50–60	60–70	More than 70
Diseases of the genitourinary system	737.7	757.3	690.5	526.3	559.7	637.7
Diseases of the circulatory system	87.4	112.0	264.3	473.7	786.0	1130.4
Respiratory diseases	396.2	288.4	231.0	241.5	259.3	355.1

## Data Availability

The data presented in this study are available on request from the corresponding author.
